# Supplementary material for: PoreVision: A Program for Enhancing Efficiency and Accuracy in SEM Pore Analyses of Gels and Other Porous Materials
Source: Gels. 2025 Feb 13;11(2):132. doi: 10.3390/gels11020132 (PMC11855315; doi:10.3390/gels11020132)
Supplement: Supplementary file 1 [file gels-11-00132-s001.zip › ImageJ Protocol.pdf]

ImageJ

Setup

<table>
<tr><td>1.</td><td>Open ImageJ</td><td>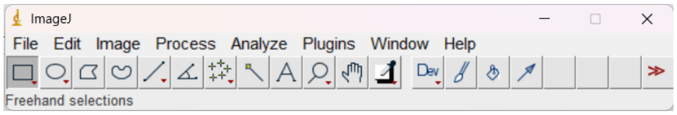</td></tr>
<tr><td>2.</td><td>Click ‘File’ → ‘Open’</td><td>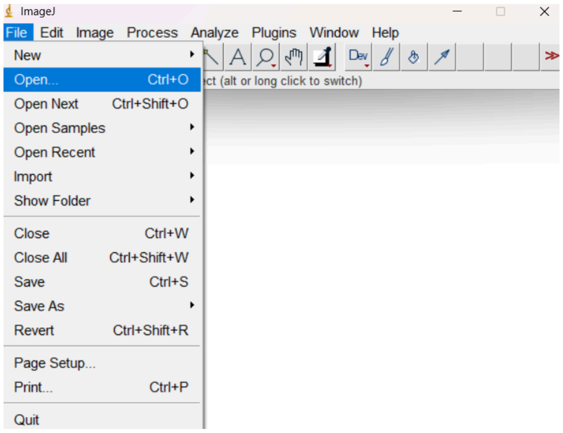</td></tr>
<tr><td>3.</td><td>Select cryogel image</td><td>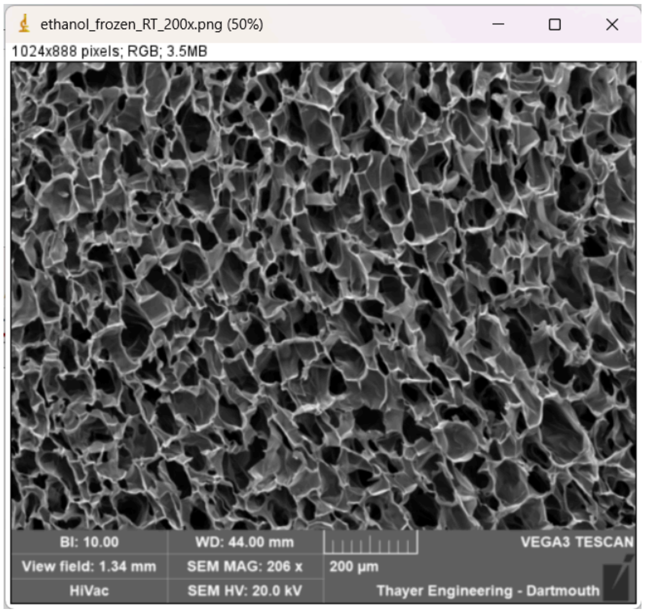</td></tr>
</table>

4.	Zoom in so that the 200 um scale bar is as large as possible on your screen<br><br>Use the hand tool to move the picture if needed	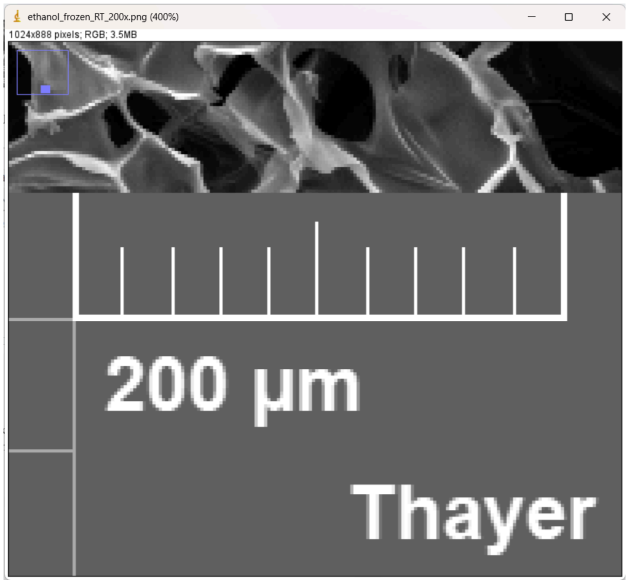
5.	Click the line tool<br>5th from the left	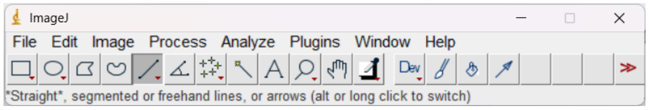
6.	Click and hold ‘shift’ to draw a straight line on top of the scale bar	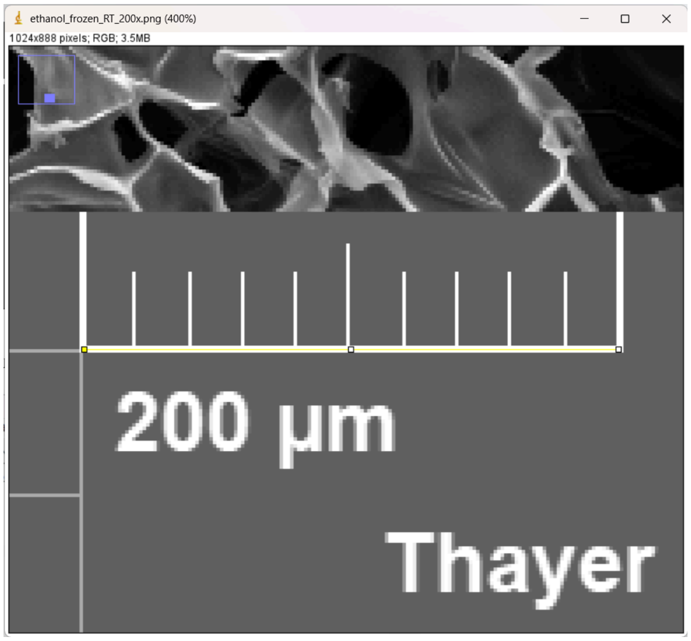

<table>
<tr>
<td>7.</td>
<td>Click ‘Analyze’ → ‘Set Scale’</td>
<td>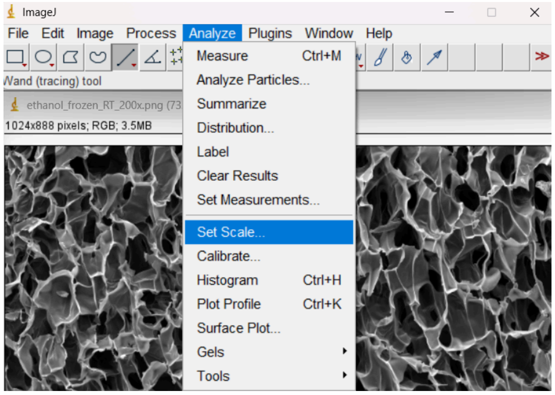
</td>
</tr>
<tr>
<td>8.</td>
<td>In the Set Scale window:

‘Distance in pixels’ should automatically populate and be about 145-165 pixels
Set ‘Known distance’ to 200
In ‘Unit of length’ type ‘micron’
‘Scale:’ should now read about 0.725-0.825 pixels/micron

There might be variation from person to person, that’s okay!

Click ‘OK’</td>
<td>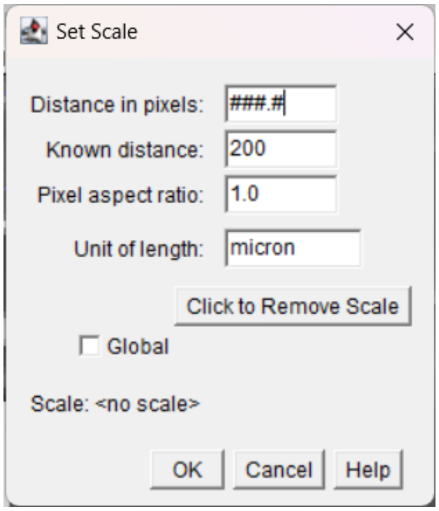
</td>
</tr>
<tr>
<td>11.</td>
<td>Click ‘Analyze’ → ‘Set Measurements…’</td>
<td>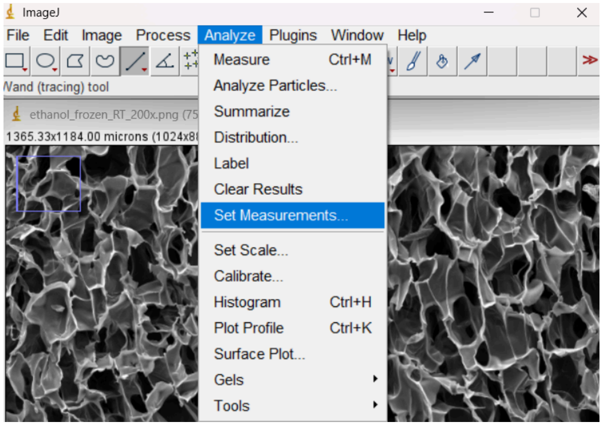
</td>
</tr>
</table>

<table>
<tr>
<td>12.</td>
<td>Tick the following boxes:

- ‘Area’
- ‘Skewness’
- ‘Perimeter’
- ‘Feret’s diameter’

Click ‘OK’</td>
<td>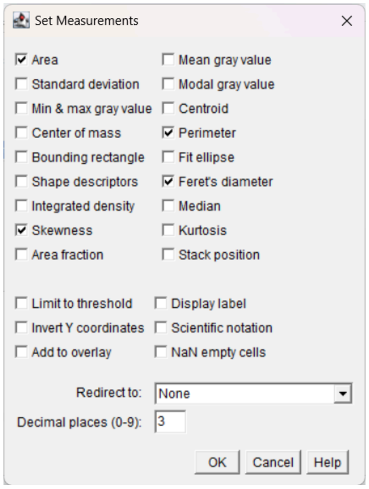
</td>
</tr>
<tr>
<td>14.</td>
<td>Click ‘Image’ → ‘Overlay’ → ‘Labels…’</td>
<td>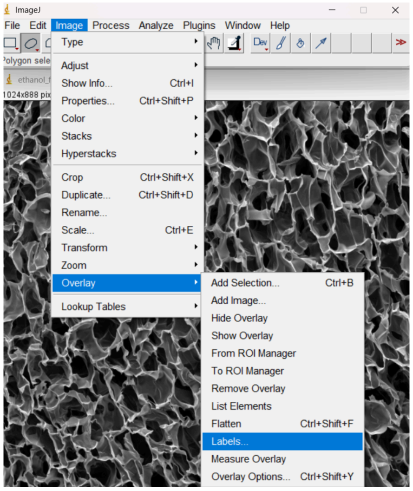
</td>
</tr>
<tr>
<td>16.</td>
<td>Change ‘Color’ to ‘yellow’

Tick the following boxes:

- ‘Show labels’
- ‘Draw backgrounds’
- ‘Bold’

Click ‘OK’</td>
<td>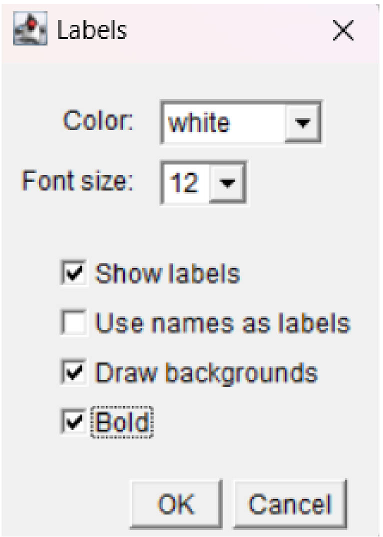
</td>
</tr>
</table>

Measurements

1.	Zoom into the top left quadrant<br><br>A blue box in the top left corner should indicate you are zoomed into a quarter of the image	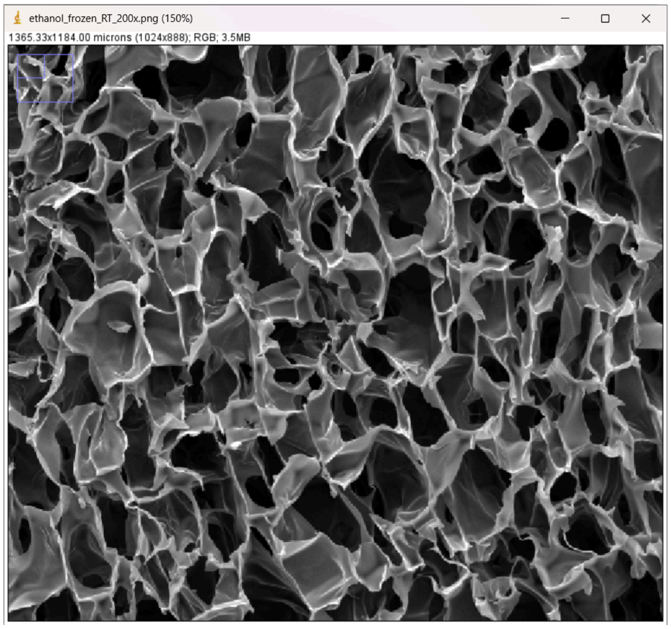<br>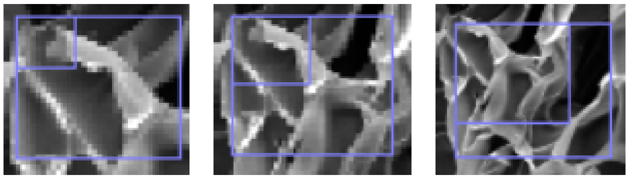<br>Less than 1/4 1/4 More than 1/4
2.	Long press the ellipse tool (2nd from the left)Click ‘Elliptical selections’	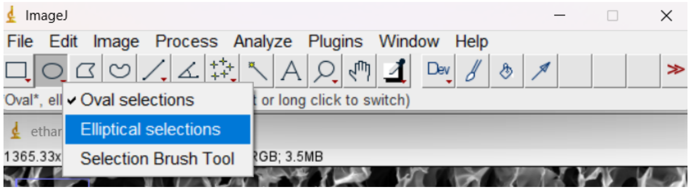

<table>
<tr>
<td>3.</td>
<td>Find a pore and create an ellipse around it

a. Use the nodes to increase or decrease the size and to rotate the ellipse

b. Try to form the ellipse so it roughly follows the perimeter of the pore

c. For pores that aren’t easy to fit into an ellipse, use your best judgment
(that’s part of the fun!)</td>
<td>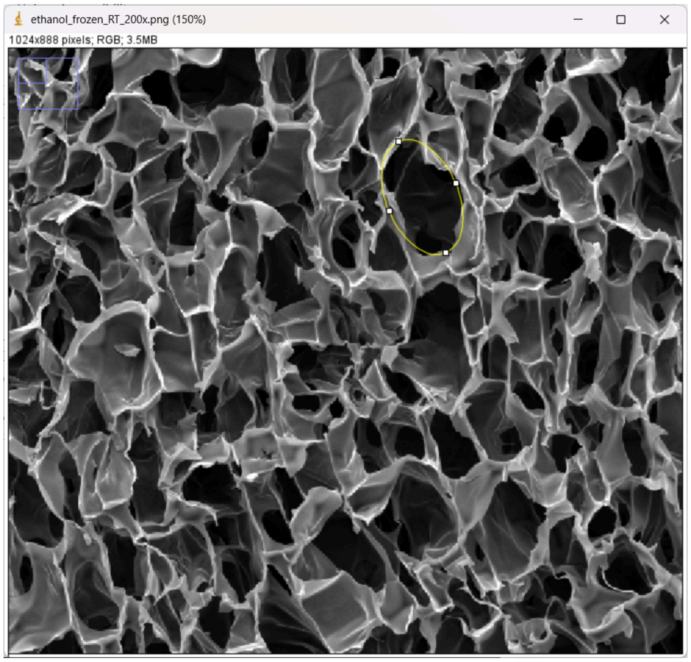
</td>
</tr>
<tr>
<td>4.</td>
<td>After you are satisfied with the ellipse:
● On your keyboard press ‘B’ (creates an imprint)
● On your keyboard press ‘M’ (adds the measurement to the list)</td>
<td>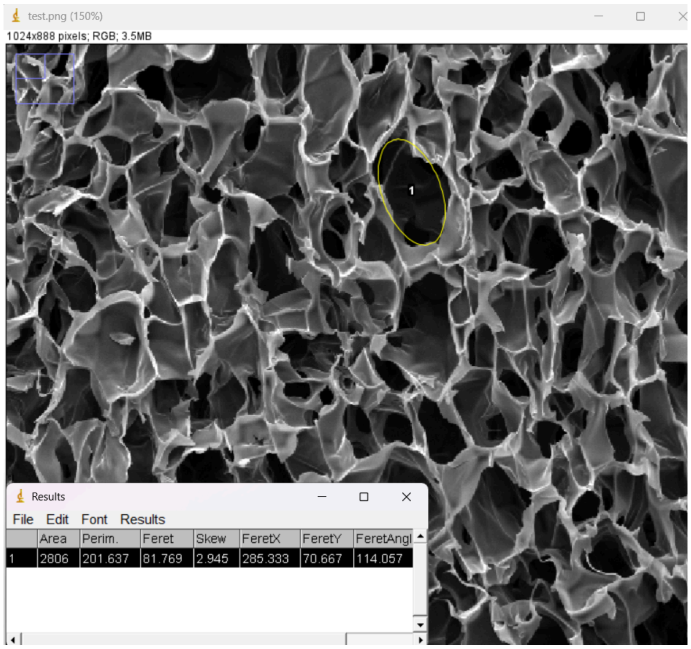
</td>
</tr>
</table>

<table>
<tr>
<td>5.</td>
<td>If you are not satisfied with the ellipse:
a. Click the row in ‘Results’ and hit delete to delete the data
b. Click the number in the middle of the ellipse to select the ellipse
c. Edit!
d. Hit ‘B’ on your keyboard
e. Hit ‘M’on your keyboard</td>
<td>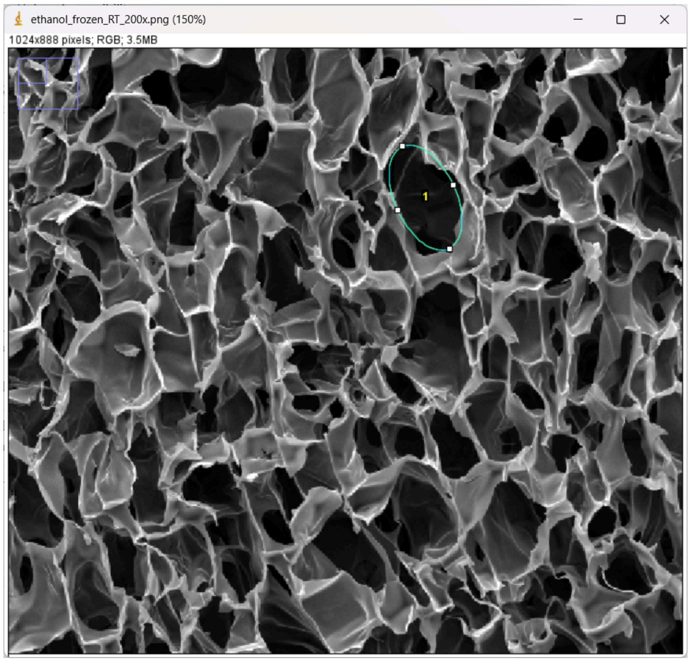
</td>
</tr>
<tr>
<td>6.</td>
<td>Continue this process until there are 15 ellipses in this quadrant

a. Aim to get a variety of ellipses (large, small, fat, skinny, etc.)

b. There should also be 15 rows in the ‘Results’ window</td>
<td>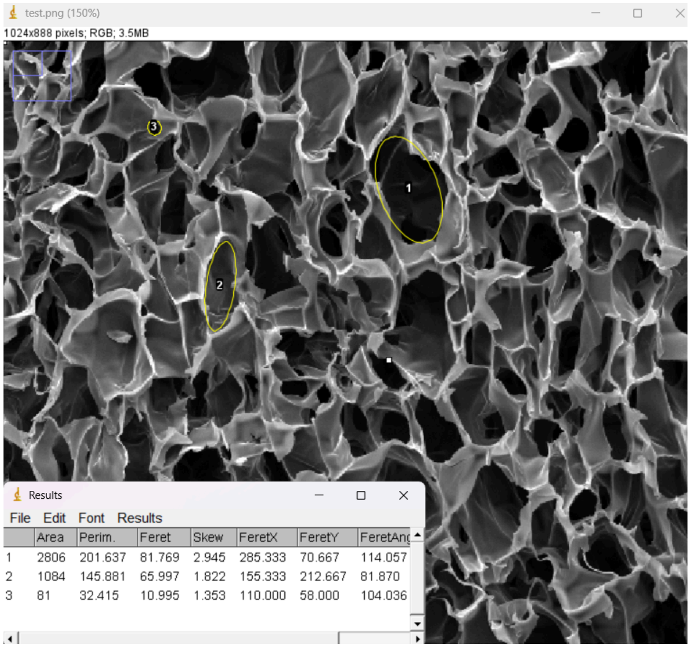
</td>
</tr>
</table>

<table>
<tr>
<td>7.</td>
<td>Repeat steps 4 through 7 for the other three quadrants

You should have 60 pores outlined and 60 rows of measurements in the ‘Results’ window</td>
<td>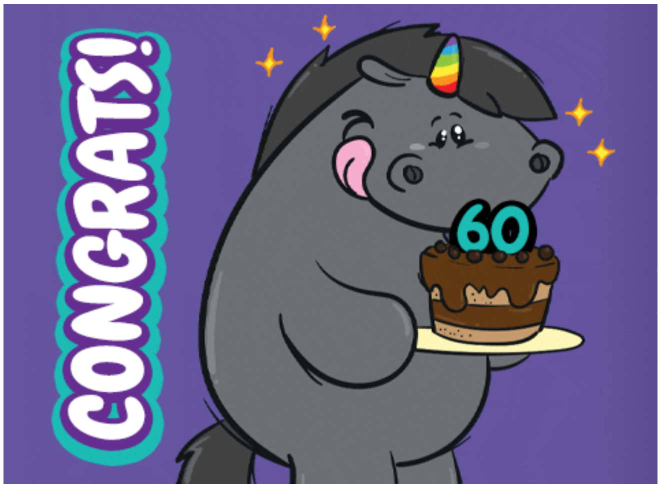
</td>
</tr>
</table>

Export

<table>
<tr>
<td>1.</td>
<td>In the ‘ImageJ’ window:
a. Click ‘File’ → ‘Save As’ → ‘PNG…’
b. Include your name and ‘ImageJ’ in the file name
c. Click ‘Save’</td>
<td>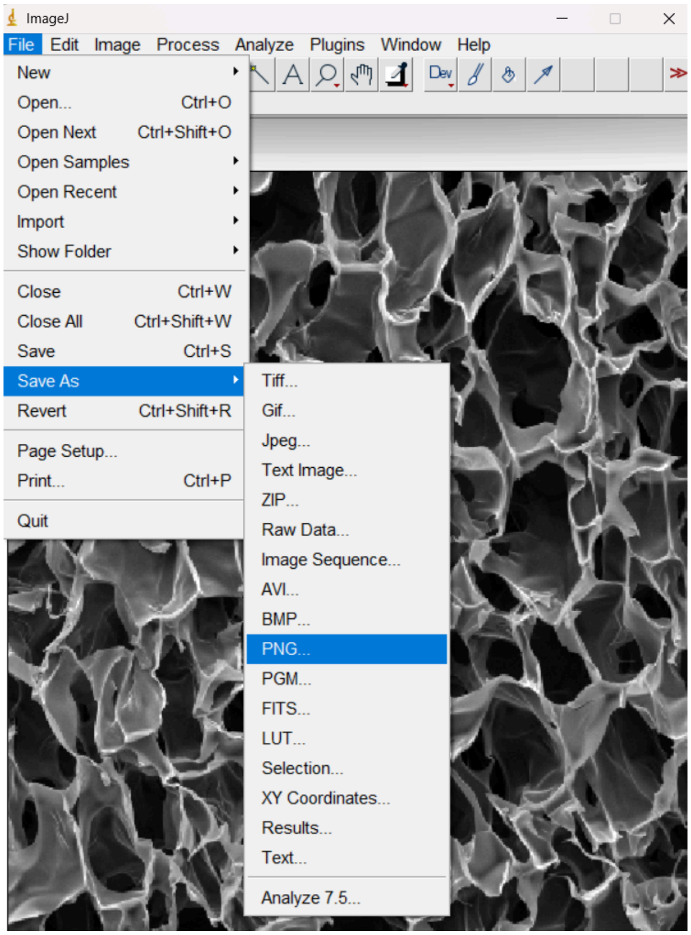
</td>
</tr>
</table>

2.	In the ‘Results’ window:<br>a. Click ‘File’ → ‘Save As…’<br>b. Include your name and ‘ImageJ’ in the file name<br>c. Click ‘Save’	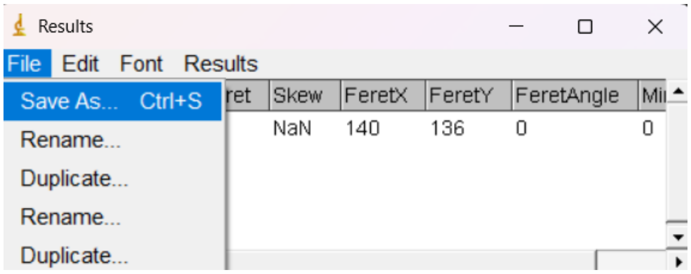
3.	Send Levi the two files by email or Slack	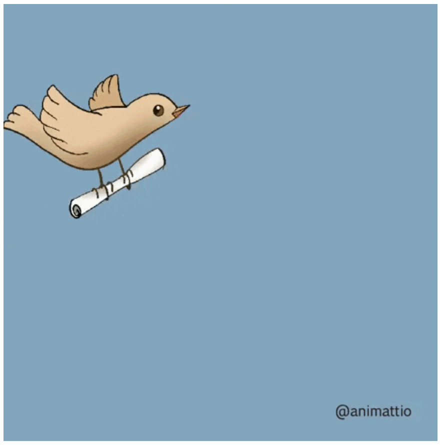
