# Supplementary material for: PoreVision: A Program for Enhancing Efficiency and Accuracy in SEM Pore Analyses of Gels and Other Porous Materials
Source: Gels. 2025 Feb 13;11(2):132. doi: 10.3390/gels11020132 (PMC11855315; doi:10.3390/gels11020132)

PoreVision Analyses of Other Types of Scaffolds

Examples of scaffold pictures with good pore morphology that can be detected with PoreVision:

Electrospun scaffold

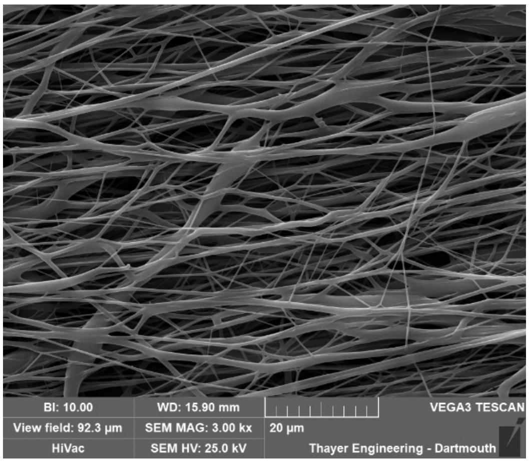

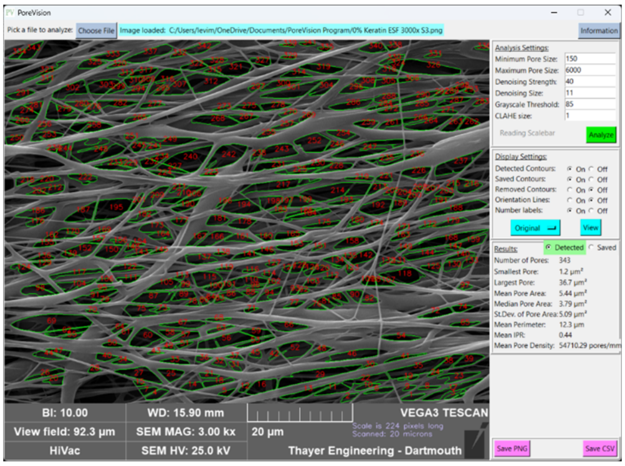

3D Printed Hydroxyapatite Scaffold

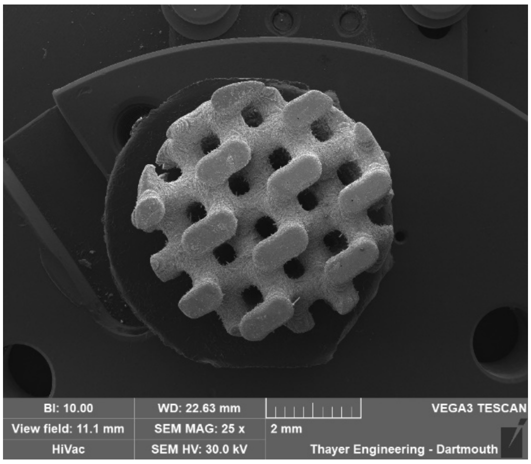

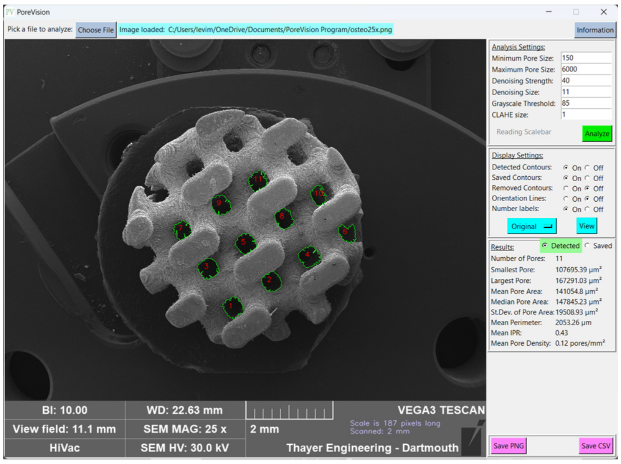

Examples of scaffold pictures with pore morphology that cannot be detected with PoreVision

Hydrogel scaffold

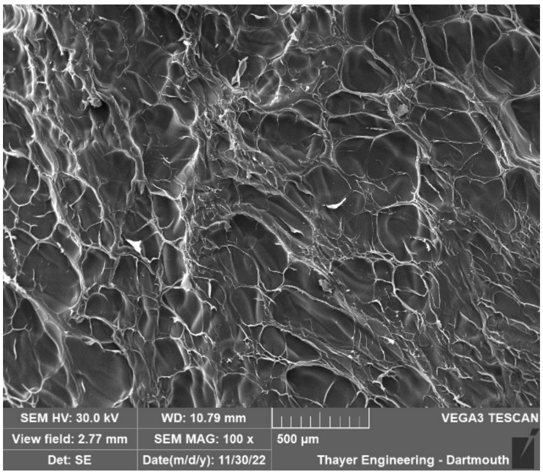

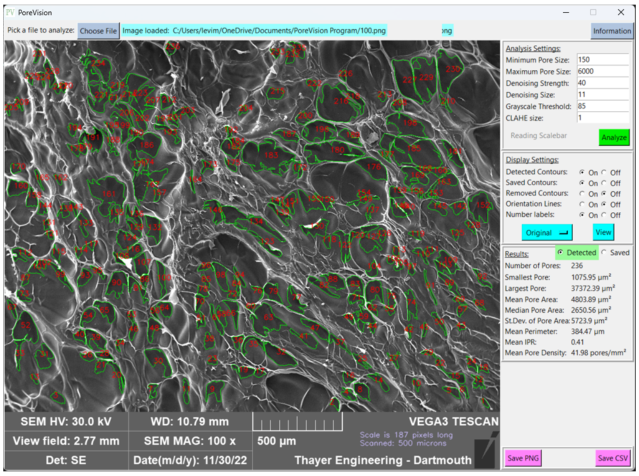

Cryogel Scaffold with poor pore formation

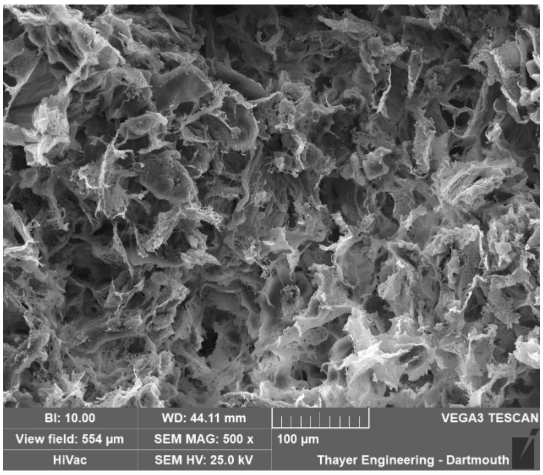

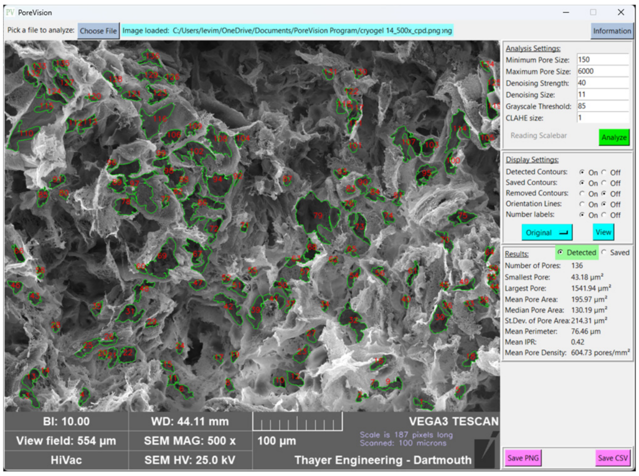

Supplement: Supplementary file 1 [file gels-11-00132-s001.zip › PoreVision Analyses of Other Types of Scaffolds.pdf]
