# Supplementary figures and images for: PoreVision: A Program for Enhancing Efficiency and Accuracy in SEM Pore Analyses of Gels and Other Porous Materials
Source: Gels. 2025 Feb 13;11(2):132. doi: 10.3390/gels11020132 (PMC11855315; doi:10.3390/gels11020132)

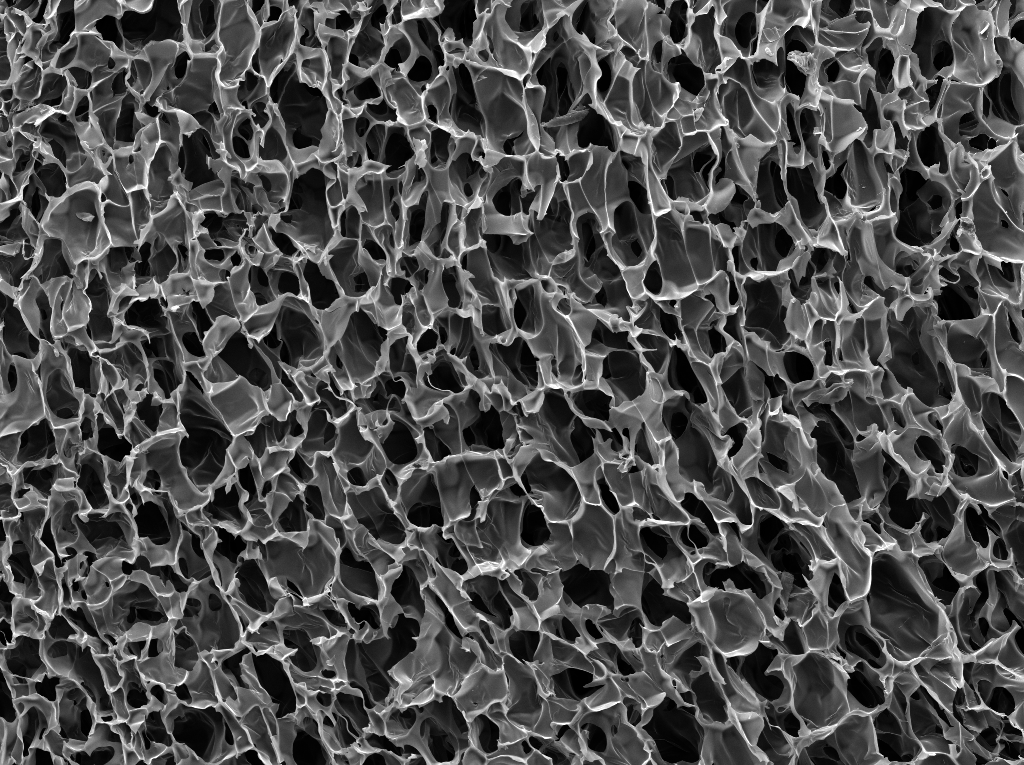

Supplement: Supplementary file 1 [file gels-11-00132-s001.zip › Pore Measurement Test Image (Cropped).png]

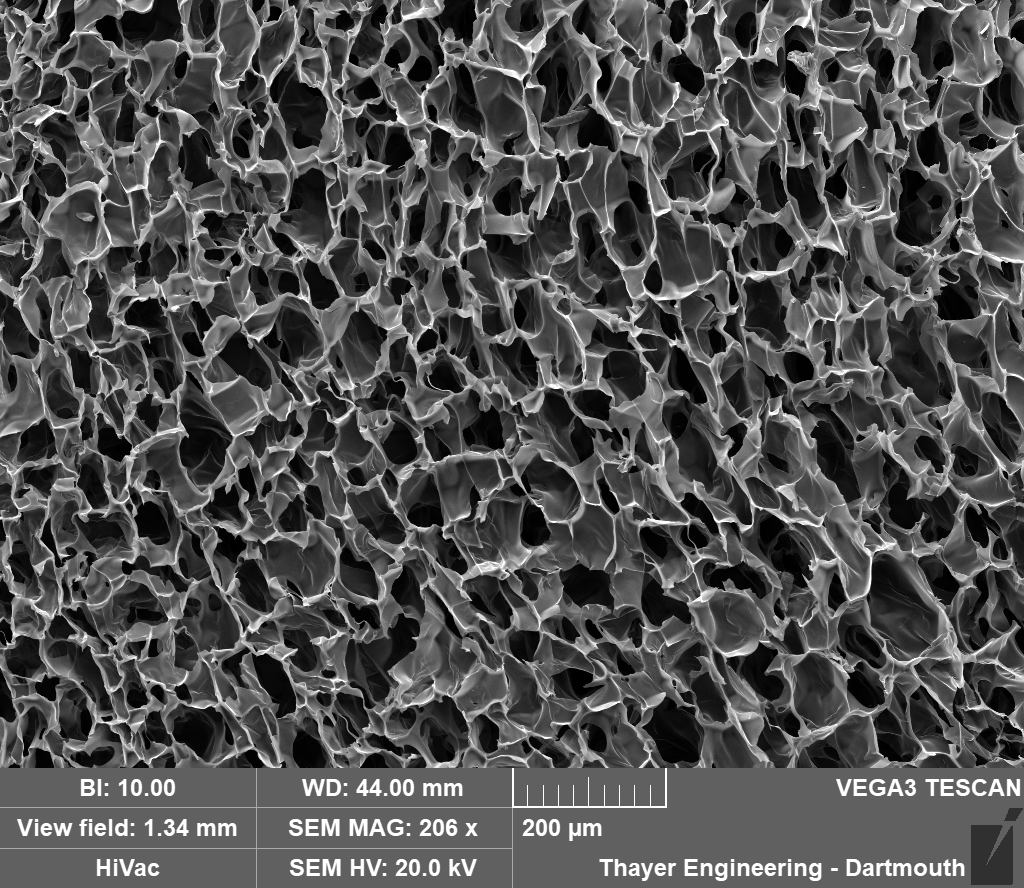

Supplement: Supplementary file 1 [file gels-11-00132-s001.zip › Pore Measurement Test Image (Original).png]

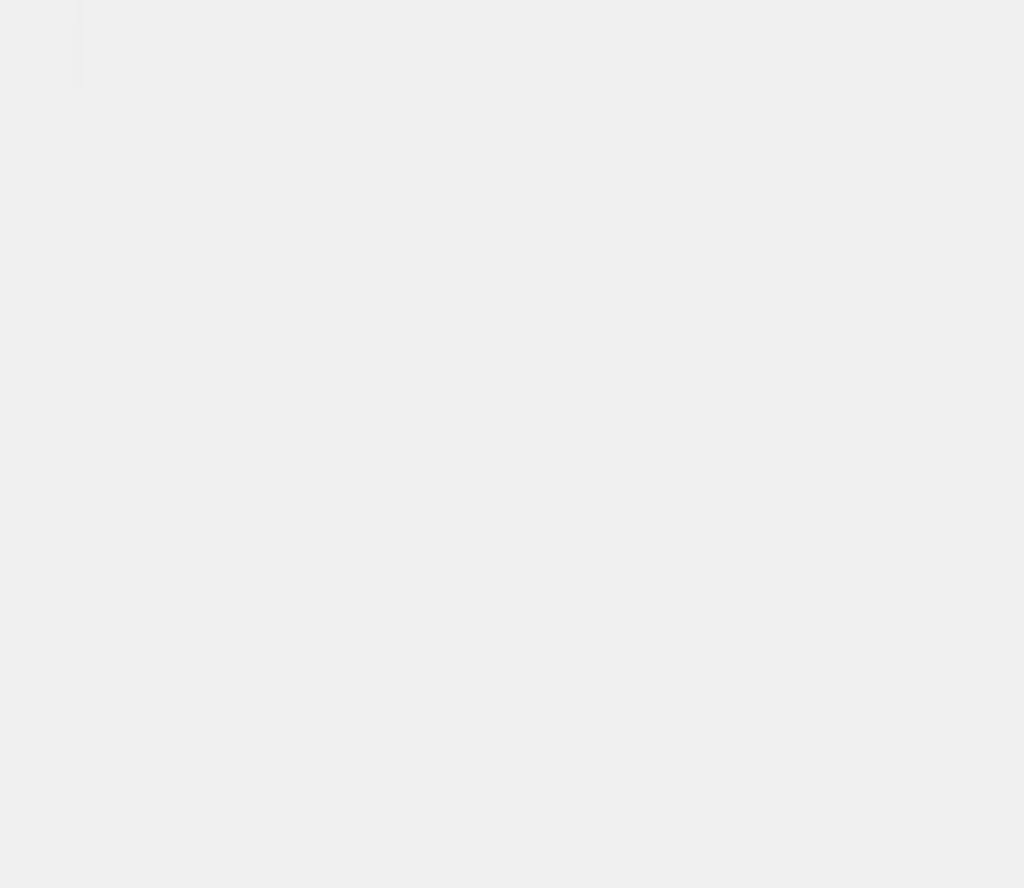

Supplement: Supplementary file 1 [file gels-11-00132-s001.zip › PoreVision Program/blank.png]

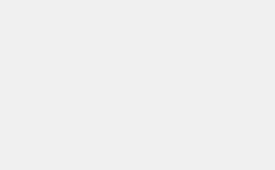

Supplement: Supplementary file 1 [file gels-11-00132-s001.zip › PoreVision Program/cover_up_indiv.png]

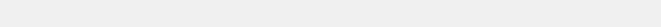

Supplement: Supplementary file 1 [file gels-11-00132-s001.zip › PoreVision Program/filepath_blocker.png]

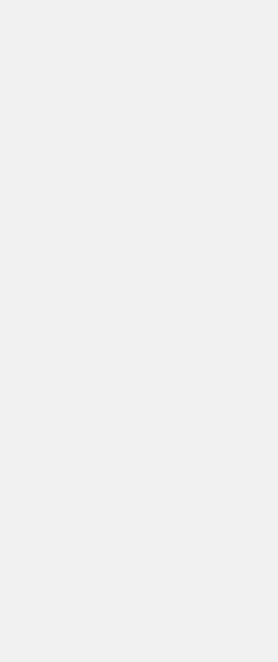

Supplement: Supplementary file 1 [file gels-11-00132-s001.zip › PoreVision Program/new_file.png]

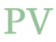

Supplement: Supplementary file 1 [file gels-11-00132-s001.zip › PoreVision Program/Pore_Program_Icon.png]

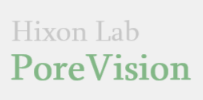

Supplement: Supplementary file 1 [file gels-11-00132-s001.zip › PoreVision Program/Pore_Program_Splash.png]

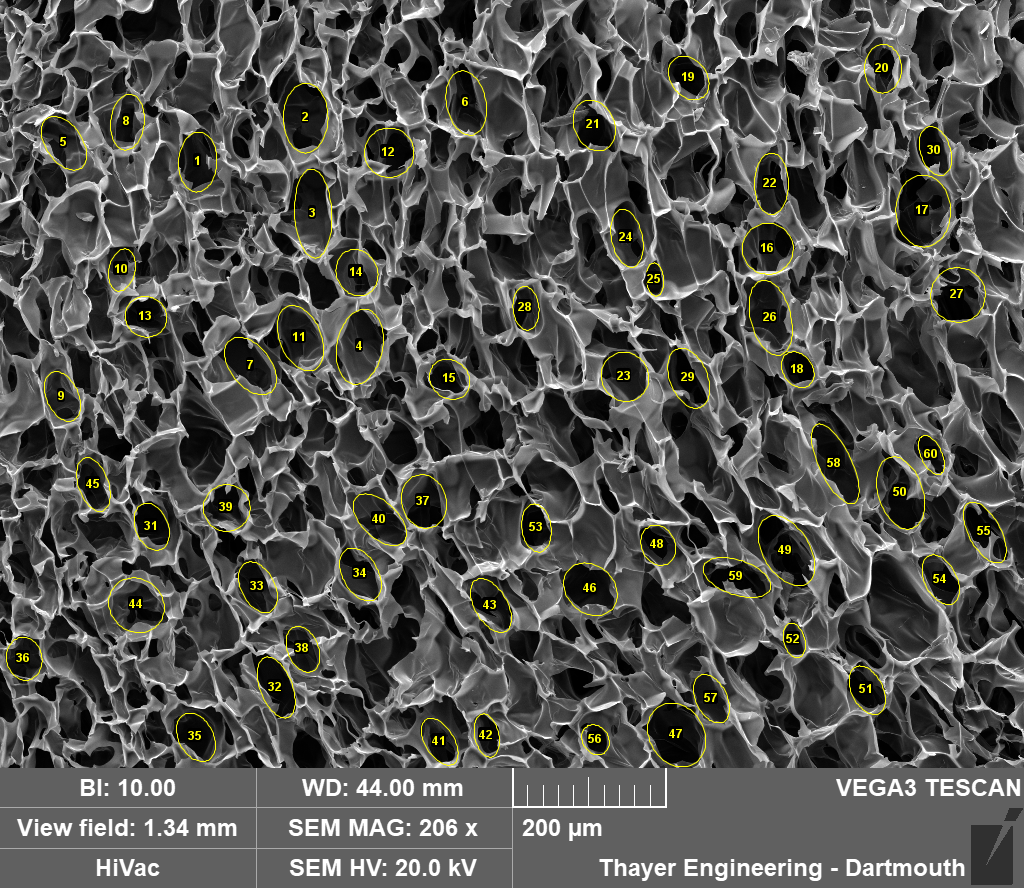

Supplement: Supplementary file 1 [file gels-11-00132-s001.zip › Testing - ImageJ/ImageJ Images/Researcher 1 - ImageJ Image.png]

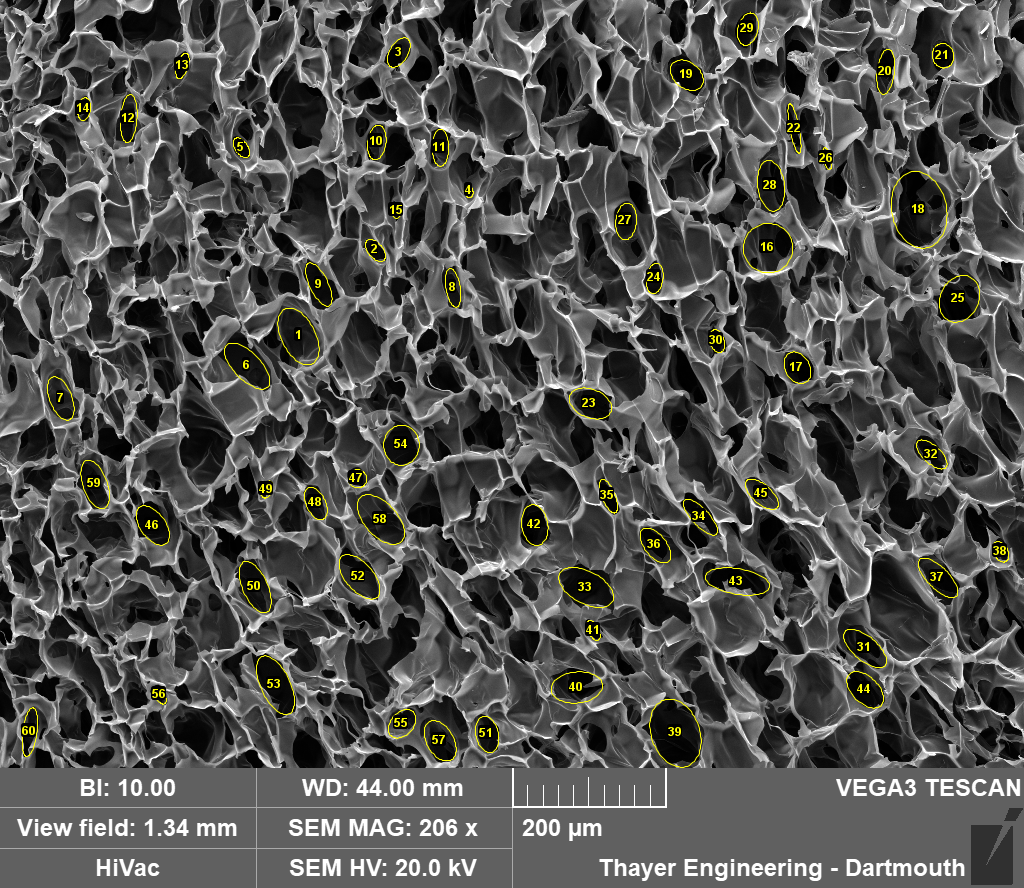

Supplement: Supplementary file 1 [file gels-11-00132-s001.zip › Testing - ImageJ/ImageJ Images/Researcher 2 - ImageJ Image.png]

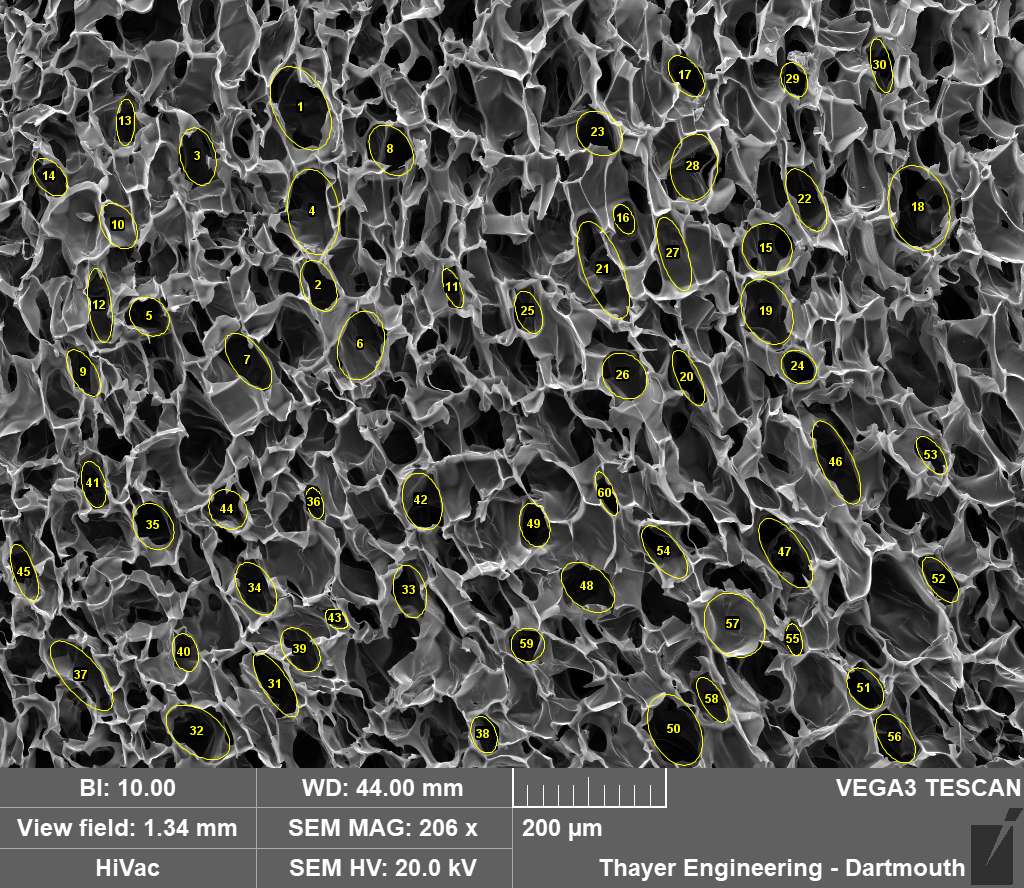

Supplement: Supplementary file 1 [file gels-11-00132-s001.zip › Testing - ImageJ/ImageJ Images/Researcher 3 - ImageJ Image.png]

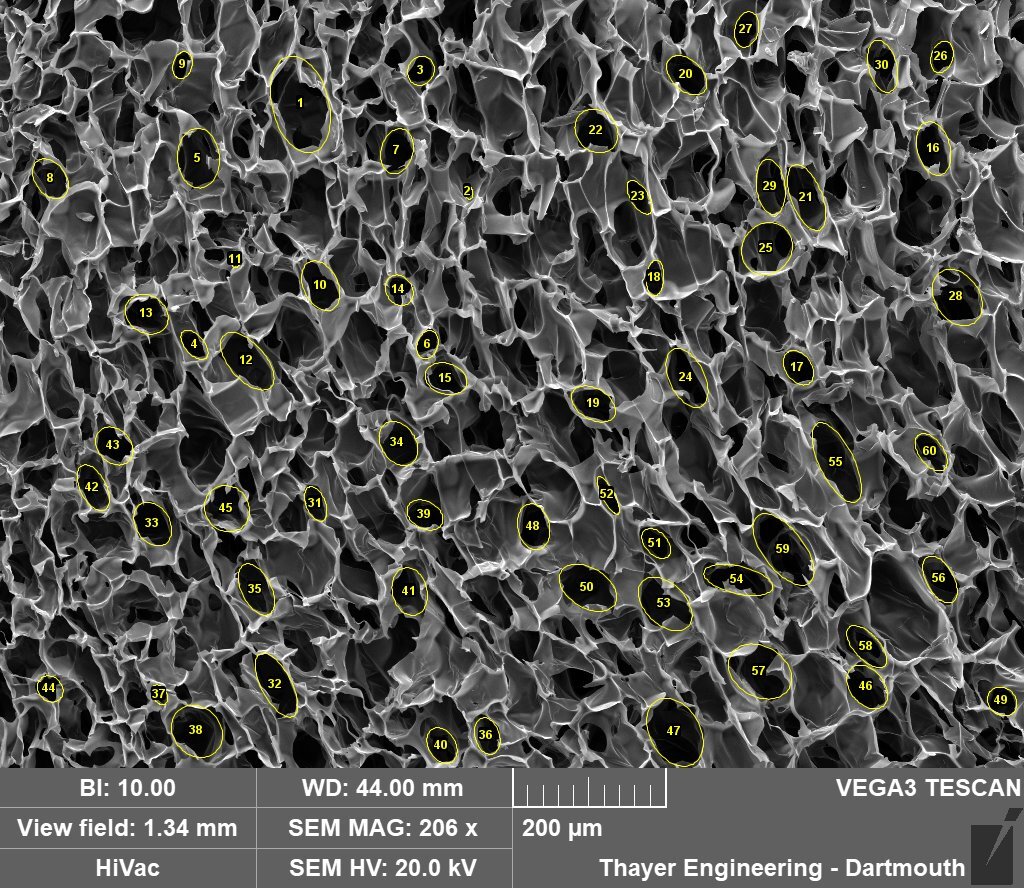

Supplement: Supplementary file 1 [file gels-11-00132-s001.zip › Testing - ImageJ/ImageJ Images/Researcher 4 - ImageJ Image.jpg]

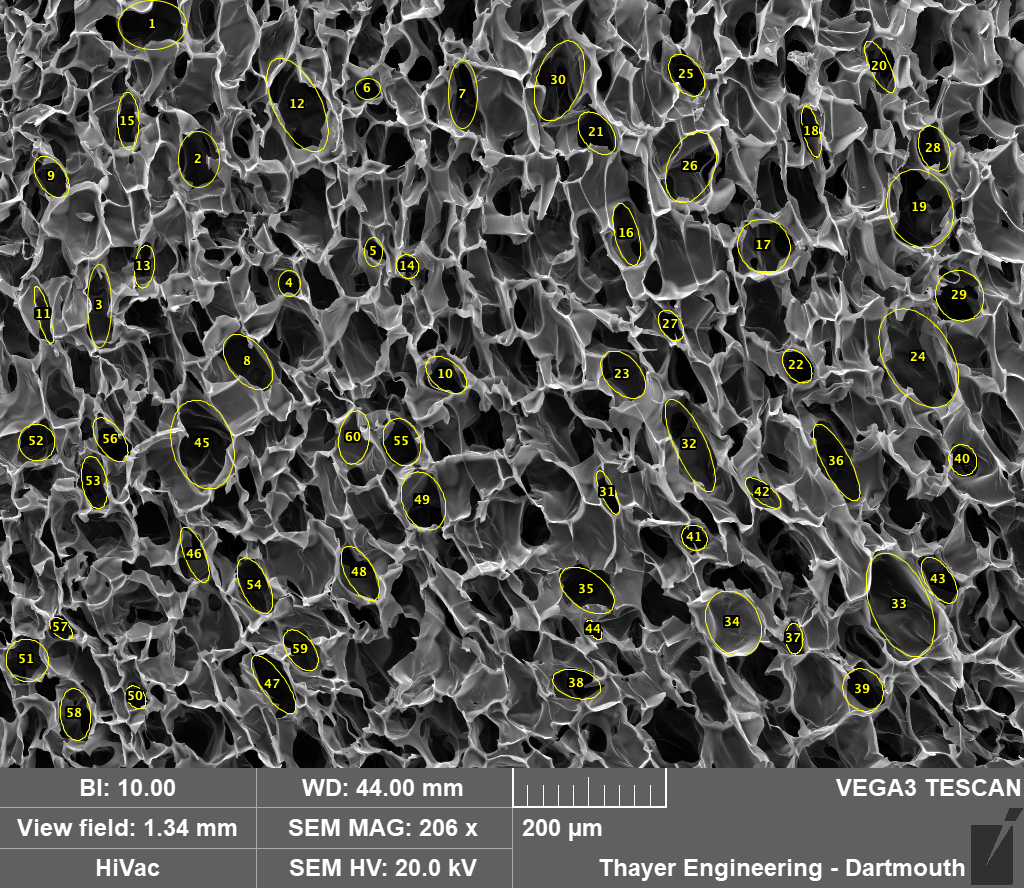

Supplement: Supplementary file 1 [file gels-11-00132-s001.zip › Testing - ImageJ/ImageJ Images/Researcher 5 - ImageJ Image.png]

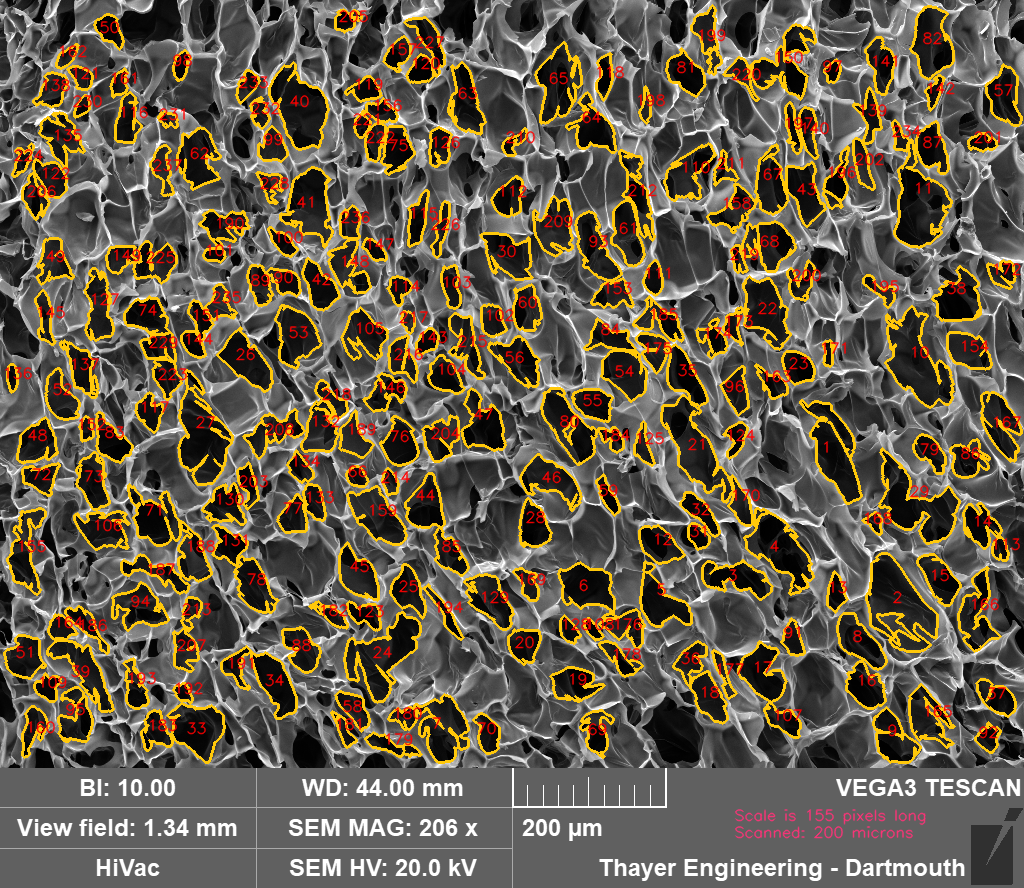

Supplement: Supplementary file 1 [file gels-11-00132-s001.zip › Testing - PoreVision/PoreVision Images/Researcher 1 - PoreVision Image.png]

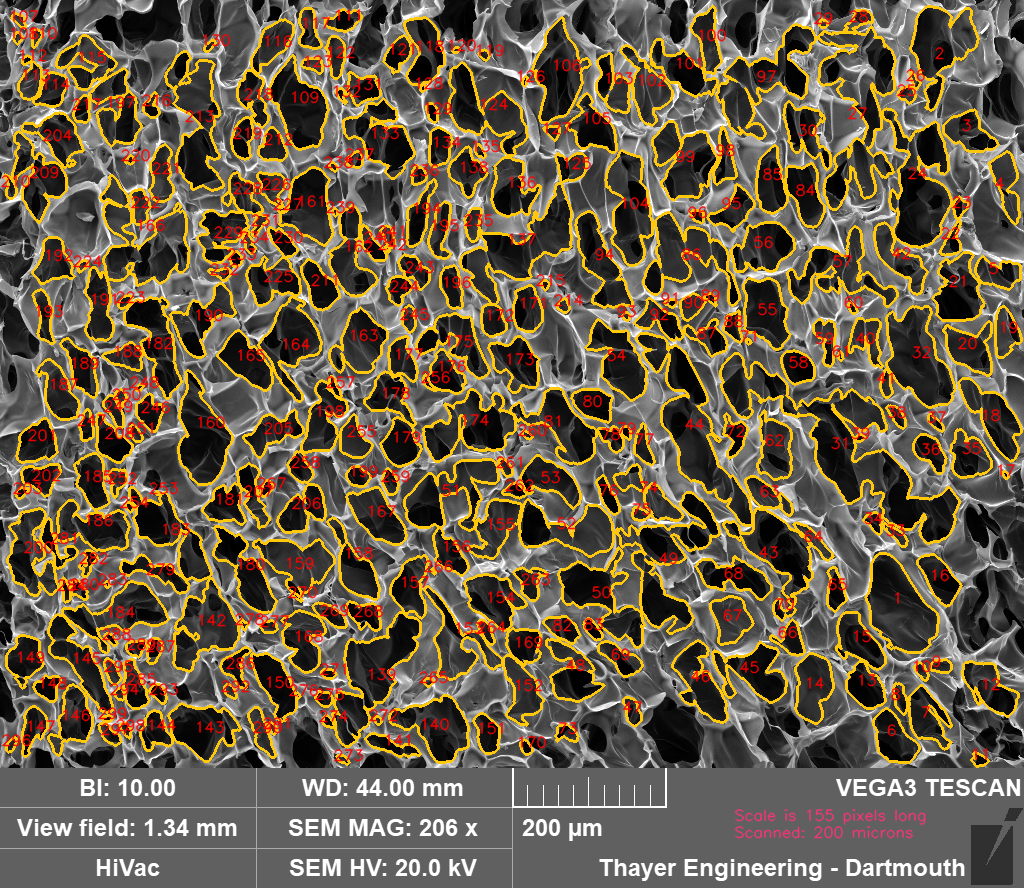

Supplement: Supplementary file 1 [file gels-11-00132-s001.zip › Testing - PoreVision/PoreVision Images/Researcher 2 - PoreVision Image.png]

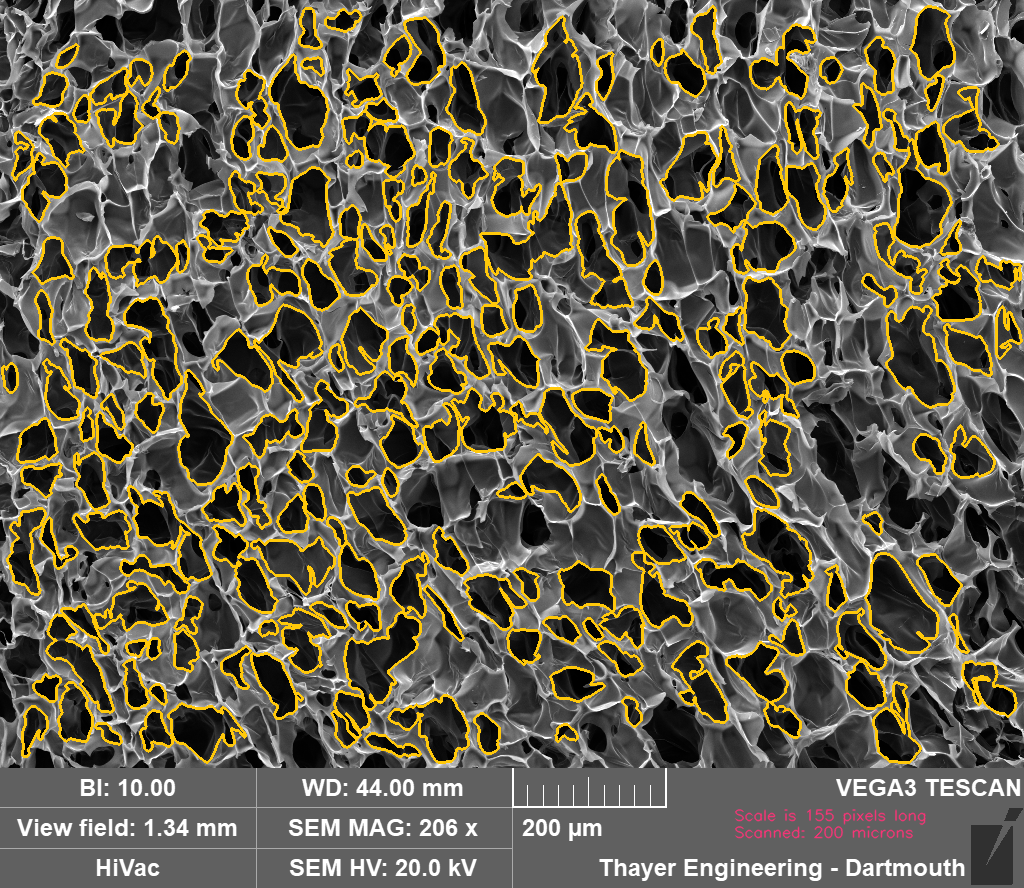

Supplement: Supplementary file 1 [file gels-11-00132-s001.zip › Testing - PoreVision/PoreVision Images/Researcher 3 - PoreVision Image.png]

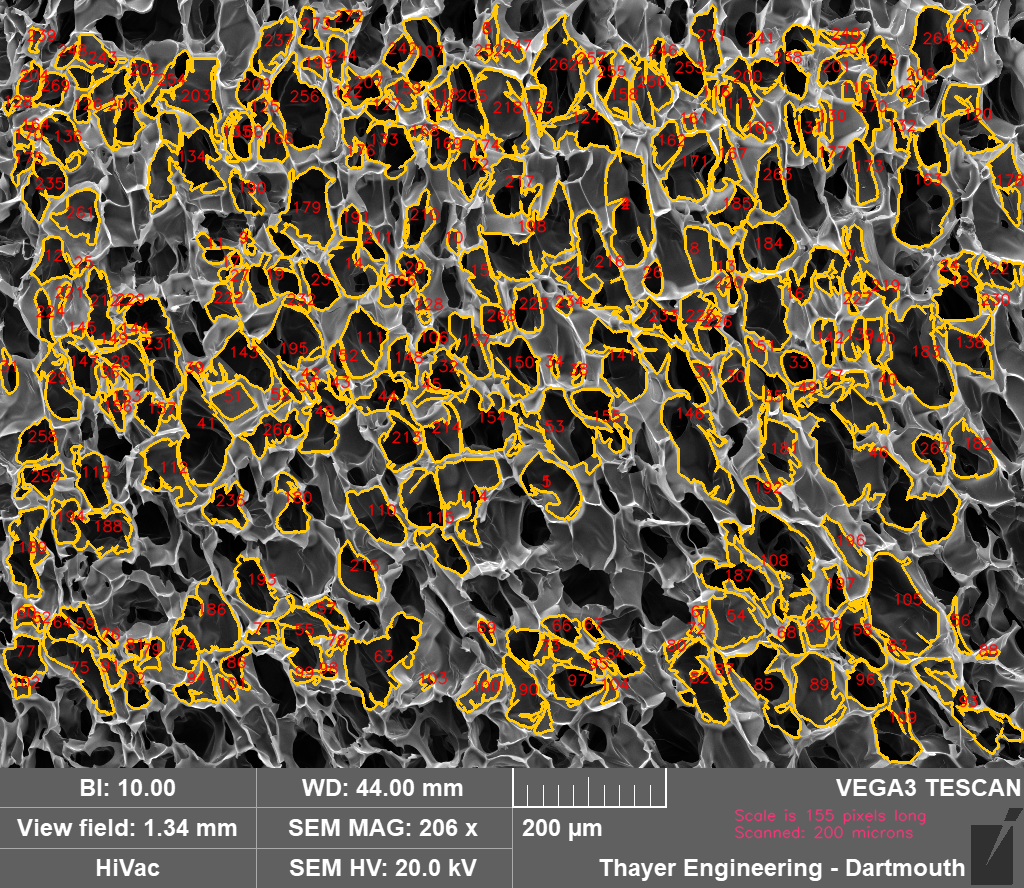

Supplement: Supplementary file 1 [file gels-11-00132-s001.zip › Testing - PoreVision/PoreVision Images/Researcher 4 - PoreVision Image.png]

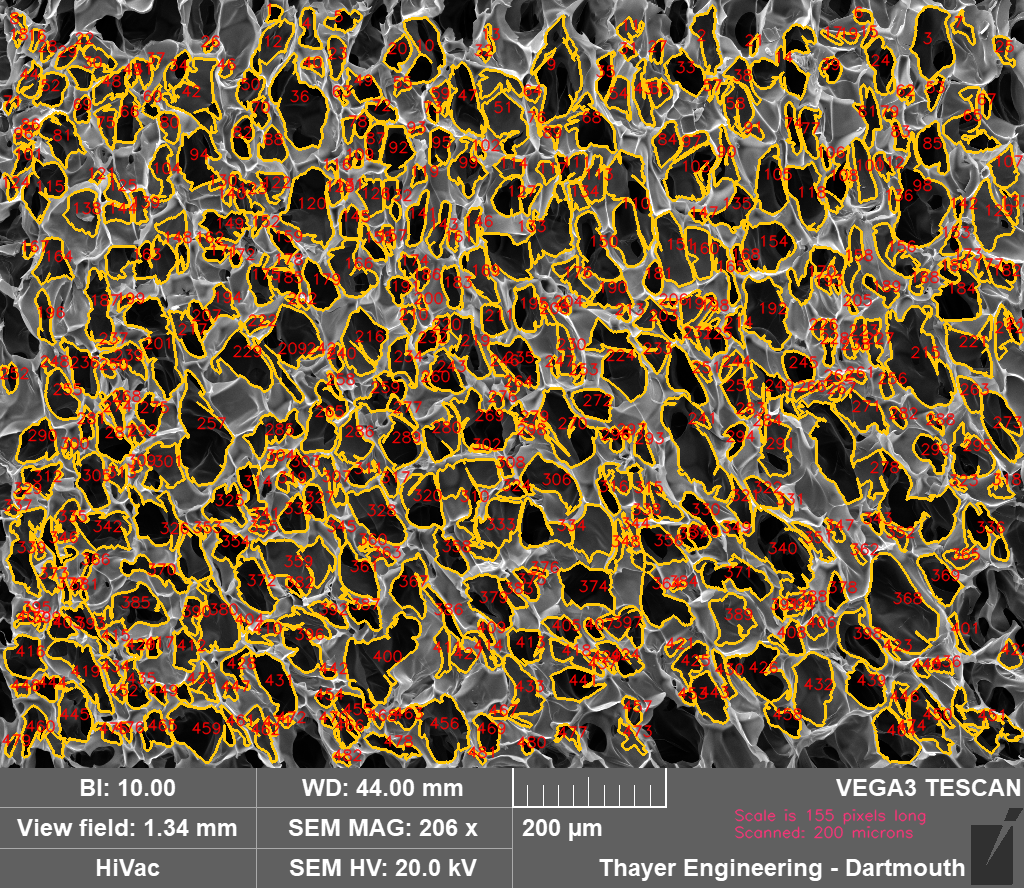

Supplement: Supplementary file 1 [file gels-11-00132-s001.zip › Testing - PoreVision/PoreVision Images/Researcher 5 - PoreVision Image.png]
